# Retraction: Interpretable machine learning framework for predicting Urban air quality

**DOI:** 10.1371/journal.pone.0342214

**Published:** 2026-02-04

**Authors:** The PLOS One Editors

Following the publication of this article [1], concerns were raised about the content of the article and the article’s peer-review. Specifically, this article discusses an interpretable machine learning framework for predicting urban air quality, but several sections of this article appear to discuss methodology and results pertaining to an unrelated study on the topic of Alzheimer’s disease. In reviewing this matter, PLOS identified additional concerns regarding the article’s peer review and compliance with PLOS’ policy on Artificial Intelligence Tools and Technologies.

PLOS regrets that these issues were not addressed prior to the article’s publication.

In light of the above concerns, which call into question the validity and reliability of the published results, the *PLOS One* Editors retract this article.

All authors did not agree with the retraction.
